# Indole-3-carbinol attenuates lipopolysaccharide-induced acute respiratory distress syndrome through activation of AhR: role of CCR2+ monocyte activation and recruitment in the regulation of CXCR2+ neutrophils in the lungs

**DOI:** 10.3389/fimmu.2024.1330373

**Published:** 2024-03-26

**Authors:** Bryan Latrell Holloman, Kiesha Wilson, Alkeiver Cannon, Mitzi Nagarkatti, Prakash S. Nagarkatti

**Affiliations:** Nagarkatti Laboratory, University of South Carolina School of Medicine, Department of Pathology, Microbiology, and Immunology, Columbia, SC, United States

**Keywords:** ARDS, LPS, CCR2, lung, inflammation, CXCL3, aryl hydrocarbon receptor, indole-3-carbinol

## Abstract

**Introduction:**

Indole-3-carbinol (I3C) is found in cruciferous vegetables and used as a dietary supplement. It is known to act as a ligand for aryl hydrocarbon receptor (AhR). In the current study, we investigated the role of AhR and the ability of I3C to attenuate LPS-induced Acute Respiratory Distress Syndrome (ARDS).

**Methods:**

To that end, we induced ARDS in wild-type C57BL/6 mice, Ccr2gfp/gfp KI/KO mice (mice deficient in the CCR2 receptor), and LyZcreAhRfl/fl mice (mice deficient in the AhR on myeloid linage cells). Additionally, mice were treated with I3C (65 mg/kg) or vehicle to investigate its efficacy to treat ARDS.

**Results:**

I3C decreased the neutrophils expressing CXCR2, a receptor associated with neutrophil recruitment in the lungs. In addition, LPS-exposed mice treated with I3C revealed downregulation of CCR2+ monocytes in the lungs and lowered CCL2 (MCP-1) protein levels in serum and bronchoalveolar lavage fluid. Loss of CCR2 on monocytes blocked the recruitment of CXCR2+ neutrophils and decreased the total number of immune cells in the lungs during ARDS. In addition, loss of the AhR on myeloid linage cells ablated I3C-mediated attenuation of CXCR2+ neutrophils and CCR2+ monocytes in the lungs from ARDS animals. Interestingly, scRNASeq showed that in macrophage/monocyte cell clusters of LPS-exposed mice, I3C reduced the expression of CXCL2 and CXCL3, which bind to CXCR2 and are involved in neutrophil recruitment to the disease site.

**Discussion:**

These findings suggest that CCR2+ monocytes are involved in the migration and recruitment of CXCR2+ neutrophils during ARDS, and the AhR ligand, I3C, can suppress ARDS through the regulation of immune cell trafficking.

## Graphical Abstract

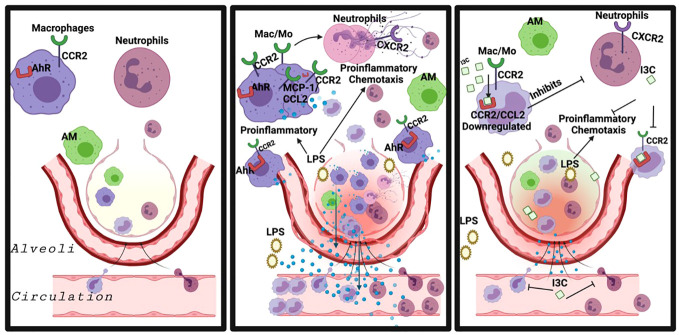

## 1 Introduction

Acute Respiratory Distress Syndrome (ARDS) is a form of respiratory failure that is caused by a variety of insults, including pneumonia, sepsis, trauma, and COVID-19 (1). It is estimated that ~33% of hospitalized COVID-19 patients develop ARDS and nearly 75% of such patients admitted to the ICU have ARDS (2). In patients, the severity of the disease is characterized by a systematic hyperimmune response, a cytokine storm, lung structure remodeling, decreased lung functionality, and systematic oxygen deprivation (3–6). While the ARDS caused by bacteria and viruses may differ in terms of the types of cells infected in the lungs, inflammation that ensues the infection can further cause significant damage to the lungs (1).

Severe damage to the lungs induced by ARDS results in severe hypoxemia and pulmonary edema. While there is no cure for this disease, significant advancements have been made in managing the symptoms and alleviating fatalities. The focus is on supportive care, targeted pharmacological interventions, and lung-protective ventilation strategies. Recent studies have illustrated the effectiveness of interventions like fluid management in improving oxygenation, neuromuscular blockade, and prone positioning, which help to improve mortality rates of ARDS patients (7, 8). Additionally, emerging therapies like extracorporeal membrane oxygenation (ECMO) have shown promise in cases of refractory hypoxemia, offering a potential lifeline for critically ill patients (9).

LPS-induced ARDS is one of the most commonly used rodent models to study ARDS (10, 11). ARDS in LPS-challenged mice is accompanied by lung remodeling, disrupted lung functionality, systematic oxygen deprivation, and a hyperimmune response (12–19). LPS-induced ARDS is a neutrophil-dependent lung injury model in which the progression of the disease depends on the activation and transmigration of neutrophils (11, 20). The sequence of neutrophil emigration to the lungs begins with blood neutrophil recruitment to the interstitium, followed by transepithelial migration to alveolar airspace (20). While for host defense and bacteria clearance, neutrophil recruitment into the lungs is necessary, the overzealous activation and accumulation of neutrophils causes damage to the lung tissue. Furthermore, neutrophil recruitment, the first leukocytes to migrate to the alveolus during lung injury, is speculated to be dependent on the activation of monocytes and macrophages in the lungs (21–24).

There are two main subsets of macrophages in the alveolus: residential alveolar macrophages (RAM) and recruited alveolar macrophages (RecAM). RecAM that migrate from the blood, also referred to as peripheral blood monocytes, are suggested to play a role in the systemic inflammatory response and pathogenesis of LPS-induced ARDS. At the same time, RAM has an M2 phenotype and is considered to be immunosuppressive (21, 25–29). During ARDS, peripheral blood monocytes are recruited to the alveolar sac, where they differentiate into recruited alveolar macrophages with an M1 phenotype (proinflammatory macrophage subset) (25, 30). Neutrophils, transmigration is reported to be dependent on proinflammatory alveolar macrophages (AM) during ARDS (20, 21). The proinflammatory macrophage subset is universally known to have an M1 phenotype. Interestingly, as previously stated, the proinflammatory macrophage subset is suspected to be recruited to the alveolus during ARDS, which leads to the controversy surrounding the question of whether neutrophils or the macrophages are the first cells to be recruited to the lungs during ARDS.

Monocytes, a type of white blood cell derived from the bone marrow, play a pivotal role in immune surveillance and inflammatory responses. These cells exhibit remarkable plasticity, transitioning between circulating monocytes and tissue-resident macrophages in various organs, including the lungs. Pulmonary monocytes are integral components of the lung’s immune defense system, contributing to both homeostasis and pathological processes such as acute lung injury and infection. Recent research has shed light on the diverse functions of pulmonary monocytes, highlighting their involvement in orchestrating immune responses, resolving inflammation, and promoting tissue repair within the lung microenvironment (31, 32). Furthermore, studies have elucidated the heterogeneity of pulmonary monocyte subsets, each with distinct phenotypic and functional characteristics, underscoring their complex role in lung immunity and pathology (33, 34). Understanding the dynamics of monocyte recruitment, activation, and differentiation within the pulmonary milieu is crucial for deciphering the mechanisms underlying lung diseases and devising targeted therapeutic interventions.

Peripheral blood monocytes entering the bloodstream during an inflammatory state express high levels of CCR2 (35, 36). CCR2+ monocytes egress and accumulate in the alveolus during LPS-induced ARDS which is dependent on CCL2 gradients in the inflamed lungs and circulation. CCR2-deficient mice treated with LPS lacked the alveolar monocyte accumulation (37–39). Some studies have also illustrated that pro-inflammatory T cells co-express multiple chemokine receptors, including CCR6 and CCR2, with distinct functions. CD4+CCR6+CCR2+ cells exhibit a pathogenic Th17 signature, produce inflammatory cytokines independent of TCR activation, and efficiently migrate through endothelial cells. While CCR6 mediates firm arrest of cells on endothelial surfaces, CCR2 is essential for transendothelial migration but not for cell arrest, indicating that these activities are mutually exclusive and depend on chemokine binding to different endothelial surfaces or forming transendothelial gradients (40). On the other hand, neutrophils recruited to the inflamed lungs express CXCR2. Interestingly, in a mouse model of polymicrobial septic peritonitis, blocking CCR2 diminished the neutrophil recruitment (41, 42). Notably, several studies have aimed to prevent neutrophil recruitment and chemotaxis and excessive neutrophil-mediated tissue damage in the lungs by inhibiting the chemokine receptor CXCR2. Navarixin (MK-7123/SCH 527123), and other CXCR2 inhibitors, have been proposed as a pharmacological intervention to treat COVID-19. However, common adverse effects include nasopharyngitis, headaches, and decreased neutrophil count, making the patients susceptible to respiratory and other infections (43–46).

The aryl hydrocarbon receptor (AhR) is a basic helix-loop-helix transcription factor that has been shown to play a critical role in the regulation of immune cell differentiation and modulation when ligated by an AhR ligand (47–49). There are several classes of AhR ligands that have been studied for their therapeutic effects against inflammatory diseases, which include endogenous (FICZ), synthetic (TCDD), and natural (Indoles) AhR ligands. Interestingly, different AhR ligands induce different immunological changes (50, 51). Indole-3-carbinol (I3C), a naturally occurring AhR ligand, has been shown to exhibit anti-inflammatory properties. I3C has been shown to downregulate LPS-induced proinflammatory gene expression of CCL2, the cytokine involved in the migration of CCR2 monocytes (52).

In the current study, we examined the role of AhR in the recruitment of proinflammatory CCR2+ monocytes and CXCR2+ neutrophils. Single-cell RNA seq t-SNEs analysis identified CCR2 as a critical receptor expressed by macrophages and monocytes populations and CXCR2 as a receptor exclusively expressed by neutrophils involved in the ARDS induction. In addition, the AhR gene was expressed by CCR2 expressing cells. Interestingly, I3C decreased the accumulation of CCR2+ monocytes and CXCR2+ neutrophils in the lungs of LPS-treated mice. In addition, the I3C-mediated decrease in CXCR2+ neutrophils was dependent on the CCR2 and AhR receptors.

## 2 Materials and methods

### 2.1 Mice

Eight-week-old female C57BL/6 and Ccr2gfp/gfp KI/KO mice (JAX stock #027619) were obtained from Jackson Laboratories (Bar Harbor, ME, USA). The Ccr2gfp/gfp KI/KO mice globally knock out CCR2 on all cell types and express GFP instead. Female LyZcreAhrfl/fl on a C57BL/6 background were bred in-house. Mice were housed at the University of South Carolina, School of Medicine AAALAC-accredited animal facility (Columbia, SC), where they were kept in temperature-controlled rooms under specific pathogen-free conditions and 12-h dark/light cycles. Mice were given ad libitum access to water and a standard chow diet, and had an average weight of 18 – 20 grams.

All animal experiments were approved by The University of South Carolina Institutional Animal Care and Use Committee.

### 2.2 LPS treatment

LPS-mediated ARDS in mice was induced as described (52, 53). Briefly, mice were anesthetized with isoflurane in combination with oxygen. These mice received 5 mg/kg of LPS (Escherichia coli, serotype 055:B5; Sigma-Aldrich, St. Louis, MO) dissolved in PBS intranasally to induce ARDS. Mice were treated with vehicle control [DMSO] or I3C [65 mg/kg] three hours after LPS-exposure. After 48 hours, mice were euthanized for studies on ARDS. The dose, frequency, and route of I3C was standardized based on our previous studies (52, 53).

### 2.3 Tissue processing

Whole blood was collected from the portal vein of mice. 500 uL of whole blood was used for VetScan analysis, and the remaining blood was either used for flow cytometry analysis or was centrifuged at 8000 rpm for 8 minutes to collect serum. Serum was used to perform enzyme-linked immunosorbent assays (ELISA). Whole blood collected for flow cytometry analysis was resuspended in 10 uL EDTA and 2 mL of red blood lysis for 10 minutes and neutralized with 10 mL flow cytometry staining buffer (FACS buffer-1X PBS, 2% Fetal Bovine Serum (FBS), and 2mM EDTA). The cells were then centrifuged and resuspended in flow cytometry staining buffer. In addition, as previously described, lung mononuclear cells (MNCs) were isolated from the whole lung (4). In brief, euthanized mice were perfused with 10 mL of heparinized PBS. Next, the lungs were removed, and BALF was collected to perform ELISA. The lungs were dissociated and brought to a single-cell suspension. The lung single-cell suspension underwent red blood lysis before being passed through a 70 μM filter. Next, the cells were centrifuged and re-suspended in flow cytometry staining buffer or 10X buffer for single-cell RNA sequence analyses. The cells were counted using the TC20 Automated Cell Counter (BioRad, Hercules, CA) to check for the viability. Samples with 80% or higher viability were processed for flow-cytometry, single-cell RNA sequence analyses, and RNA extraction for qPCR.

### 2.4 Flow cytometry and sorting

For the detection of immune cells from the lungs and blood, fluorescently labeled monoclonal antibodies (mAbs) were used along with flow cytometry as described by previously (54) In brief, cells derived from the lungs (1 x 106) were incubated with TruStain FcX anti-mouse CD16/32 (Biolegend, San Diego, CA) and tagged with fluorescently labeled mAbs APC Cy7 labeled anti-CD45, AF 700 or BV785 labeled anti-CD11b, BV785 labeled anti-CD11c, BV605 labeled anti-CD11c, AF488 labeled anti-Ly6c, APC labeled anti-Ly6c, BV785 labeled anti-Ly6g, PE-labeled anti-Ly6g, BV510 labeled anti-Gr-1, AF488 labeled anti-F4/80, BV421 labeled anti-F4/80, AF647 labeled anti-CD 206, BV650 labeled anti-CD 206, Per Cp labeled anti-CCR5, FITC labeled anti-CCR2, and/or PE-labeled anti-CXCR2 purchased from Biolegend. UltraComp eBeads (Invitrogen, Waltham, MA) were used for compensation. Cells were analyzed using a BD FACSCelesta (Franklin Lakes, NJ), and FlowJo (BD Biosciences, San Jose, CA) was used to analyze FCS files.

### 2.5 RNA extraction and qPCR

As previously described (55), the remaining cells from the lungs were suspended and spun down in 700 uL of QIAzol. Samples were then stored at -80°C until isolation. Qiagen RNeasy kit was used to isolate total RNA. The Nanodrop 2000 (Waltham, MA) was used to check RNA quality and quality. Next, the miScript II RT Kit supplied by Qiagen (Germantown, MD) was used to make cDNA. The cDNA was used in combination with SsoAdvanced SYBR green supermix from Bio-Rad for the generation of mRNA. qRT-PCR was carried out on a CFX96 Touch Real-Time PCR Detection System (Bio-Rad, Hercules, CA). Gene expression levels were normalized to β-actin expression. A detailed list of primers for genes is listed in **Table 1**.

**Table 1:** Primer sequences.

Gene name	F Primer Sequence	R Primer Sequence
CCR2	GCTGTGTTTGCCTCTCTACCAG	CAAGTAGAGGCAGGATCAGGCT
CXCR2	CTCTATTCTGCCAGATGCTGTCC	ACAAGGCTCAGCAGAGTCACCA

scRNASeq was conducted as described previously (52, 56). The 10x Chromium instrument (10x Genomics, Pleasanton, California) was used to perform single-cell RNA sequencing. Cells (3000) were loaded onto the Chromium Controller [10x Genomics] that generated single-cell Gel Bead-In-Emulsions. Next, samples were processed into single-cell RNA-seq libraries using the Chromium v2 single-cell 3′ RNA-seq reagent kit [10x Genomics]. The libraries were sequenced with NextSeq 550 instrument (Illumina, San Diego, CA). Raw sequencing data were processed using Cell Ranger version 3.1.0 (10x Genomics). Downstream analysis of Cell Ranger output was completed using Loupe Browser 5.1.0. Cell cluster biomarkers were identified using differential gene expression in Loupe Browser.

### 2.6 Statistical analyses

Statistical analyses were performed using Graphpad software. All data shown in figure legends are presented as the mean ± standard error of the mean [SEM]. One-way ANOVA tests were used to calculate the statistical significance. The number of mice or samples tested were presented as individual dots in bar graphs. The level of statistical significance was determined using the following key: *p < 0.05, **p < 0.01, ***p < 0.001, ****p<0.0001.

## 3 Results

### 3.1 scRNAseq immune profiling of lung-derived cells shows that I3C administration in LPS-treated mice induces gene dysregulation associated with cell migration

**Figure 1:**
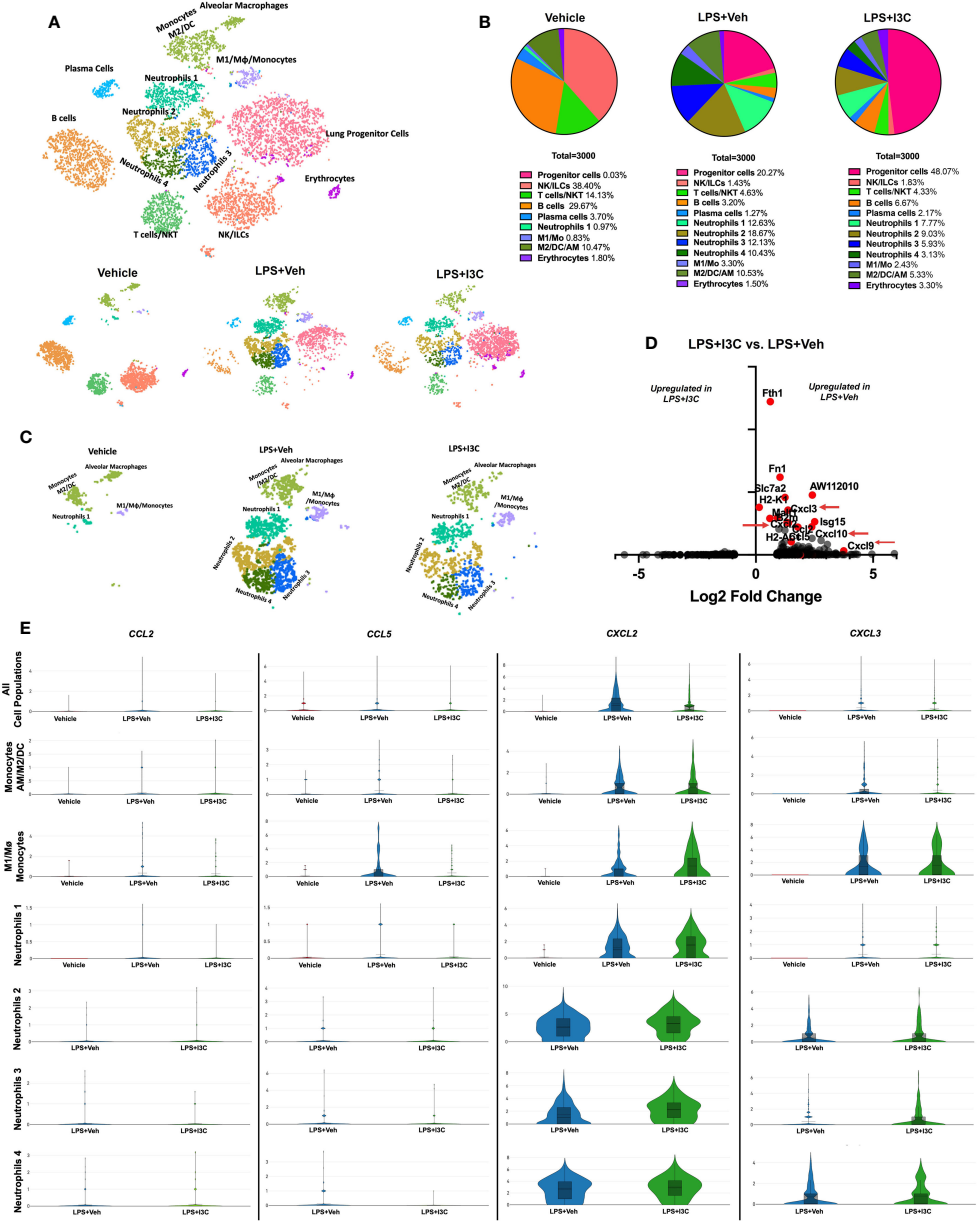
scRNAseq immune profiling of lung cells across treatment groups. Mice were exposed to LPS to induce ARDS followed by treatment with I3C as described in the Methods. The cells isolated from the lungs were screened as follows. **(A)** scRNAseq t-SNE colored by cell population. **(B)** Pie chart of cell populations by percentages. **(C)** scRNAseq t-SNE of myeloid cells. **(D)** Volcano plot of top differentiated genes in myeloid clusters in LPS+Veh *vs*. LPS+I3C samples. **(E)** Violin plots of chemokine ligand CCL2, CCL5, CXCL2, and CXCL3 gene expression across myeloid cell populations in treatment groups.

We have previously shown that I3C acts as an AhR ligand to attenuate LPS-mediated ARDS (44, 45). In the current study, we used scRNA-seq to determine lung immune cell profiles across treatment groups. Using lineage-defining genes, we identified several immune cell populations. Notably, Loupe Browser generated t-SNEs revealed considerable heterogenicity across immune cell populations in the control group *vs*. LPS+Veh and LPS+I3C groups. In LPS-challenged mice, there was an increase in clusters expressing myeloid cell gene signatures, while there was a decrease in clusters expressing lymphocyte gene signatures when compared to the control group (**Figures 1A, B**). Next, comparing the LPS+Veh group *vs*. LPS+I3C, we noticed a change in the cell profiles in the two groups (**Figures 1A, B**). Interestingly, lung progenitor cells were not present in the Vehicle-treated mice, started to appear during ARDS, and following treatment with I3C, there was a robust increase in these cells (**Figure 1B**). A further look into the myeloid cell clusters, we categorized four neutrophil populations using the clusters’ transcriptional profiles. These were designated the neutrophil subgroups Neutrophils 1, Neutrophils 2, Neutrophils 3, and Neutrophils 4 determined by cluster gene signatures (SI appendix, **Supplementary Figures 1A, B**). These clusters were markedly upregulated following LPS treatment (**Figures 1A–C**). In contrast, the vehicle-alone group expressed only a small percentage of the Neutrophil 1 cluster (0.97%) and none of the other neutrophil clusters. Moreover, LPS+I3C group showed a decrease in all 4 neutrophil clusters when compared to LPS+Veh group (**Figure 1A–C**). When we compared the proportions of M2 *vs* M1 macrophages, this ratio was highest in Vehicle controls with more M2 than M1 macrophages. This ratio was decreased following LPS treatment and was further reduced in LPS+I3C group (**Figures 1A, B**). The proportions of lymphocytes such as T/NKT cells, B cells, and plasma cells were higher in the Vehicle group and were decreased in LPS or LPS+I3C groups. The NK/ILC population was highest in the Vehicle group and markedly decreased in LPS+Veh and LPS+I3C groups.

To determine the effect of I3C on myeloid cells, we investigated the differentially expressed genes amongst LPS+Veh and LPS+I3C groups. Interestingly, genes significantly dysregulated across the two treatment groups were associated with proinflammatory responses, antigen presentation, and immune cell migration. Comparing the LPS+Veh group *vs*. LPS+I3C, we noticed I3C treatment led to downregulation of chemokine transcripts for CCL2, CCL5, CXCL2, and CXCL3 associated with chemotaxis of myeloid cells (**Figures 1D, E**). Macrophages, dendritic cells, monocytes, and neutrophils secrete CCL2 and CCL5 proteins, which are ligands that bind to CCR2+ and CCR5+ monocytes leading to the emigration of monocytes from circulation to the tissue injury site. In addition, macrophages, dendritic cells, monocytes, and neutrophils secrete CXCL2 and CXCL3 proteins, which binds to the CXCR2 on neutrophils, leading to their migration to the site of inflammation. Interestingly, CCL2, CCL5, CXCL2, and CXCL3 genes were disproportionately expressed across myeloid cell clusters in Vehicle, LPS+Veh, and LPS+I3C groups. In LPS-exposed mice, there was an increase in CCL2, CCL5, CXCL2, and CXCL3 gene expression in most of the myeloid cell clusters compared to vehicle control mice. Next, comparing the LPS+Veh group *vs*. LPS+I3C, we noticed that I3C-mediated a downshift in CCL2, CCL5, CXCL2, and CXCL3 in most cell populations (**Figure 1E**). However, especially with CXCL2 expression, some cell clusters such as M1 macrophages and neutrophil subpopulations expressed slightly higher levels of in LPS+I3C *vs* LPS+Veh group (**Figure 1E**). Together these data suggested that when all lung cells were taken into consideration, the expression of CCL2, CCL5, CXCL2, and CXCL3 was upregulated following LPS exposure and I3C treatment significantly reduced their expression. However, scRNA-seq demonstrated that individual myeloid clusters expressed differential levels of these chemokines and differential response to LPS or LPS+I3C treatment. Also, of the 4 chemokines, CXCL2 and CXCL3 expression was higher than the other two chemokines screened among the myeloid clusters. While these results may be inconsistent with what was expected, it is important to note that our understanding of the immunological landscape of the lung with and without disease will continue to change as we implement new techniques like scRNA-seq that allow us to evaluate cells with higher specificity. While this data focuses on transcript level of gene expression, there may be post-transcriptional changes that will cause changes in protein expression of these chemokines.

### 3.2 Ingenuity pathway analysis prediction of dysregulated chemoattractant ligand and receptor genes associated with CCR2+ monocytes and CXCR2+ neutrophils in LPS-exposed mice following I3C treatment

**Figure 2:**
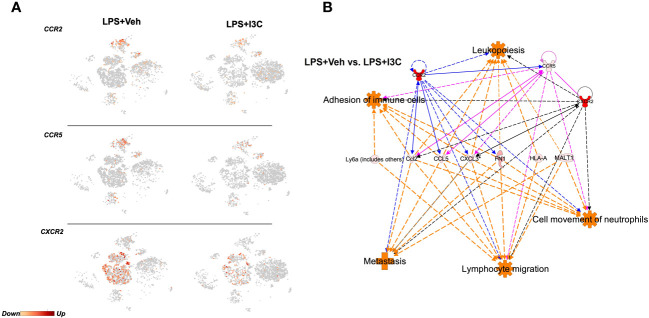
Ingenuity Pathway Analysis prediction of dysregulated chemoattractant ligand genes in LPS-exposed *vs* LPS+I3C treated mice. Mice were exposed to LPS to induce ARDS followed by treatment with I3C as described in the Methods and cells from the lungs were screened as follows. **(A)** scRNAseq t-SNE heatmap expression of CCR2, CCR5, and CXCR2. **(B)** IPA pathway prediction graphical summary of local gene dysregulation associated with immune cell trafficking functions when comparing LPS+Veh *vs*. LPS+I3C.

The recruitment of CCR2+ and CCR5+ monocytes and CXCR2+ neutrophils to the lungs during inflammation is heavily dependent on the ligand-receptor interactions. Therefore, we investigated whether the transcriptional activity of these receptors was altered in LPS-exposed mice following treatment with I3C. We found that I3C decreased the expression of CCR2, CCR5, and CXCR2 in myeloid cell clusters (**Figure 2A**). CCR2 and CCR5 are gene signatures of macrophages, monocytes, and dendritic cell clusters. On the other hand, CXCR2 is highly expressed by neutrophils (**Figure 2A**).

Next, ingenuity pathway analysis software was used to generate a pathway prediction of dysregulated genes in LPS+Veh *vs* LPS+I3C groups. This software allowed us to predict pathways due to the causal analysis algorithms utilized based on a master network derived from the Ingenuity Knowledge Base. Therefore predictions were made based on gene expression levels and validated results from previously published literature. Upon uploading our gene expression data for the most significant differentially expressed genes, we performed core analysis. Interestingly, IPA predicted dysregulated genes in LPS+Veh *vs* LPS+I3C groups to be associated with immune cell trafficking functions (**Figure 2B**). These immune cell trafficking functions included cell movement of neutrophils, lymphocyte migration, adhesion of immune cells, and metastasis. Towards this, IPA predicted CCL2, CCL5, and CXCL3 to be connected to immune cell trafficking functions. In addition, IPA predicted CCL2-CCR2/CCR5/CXCR2, CCL5-CCR2/CCR5, and CXCL3-CXCR2 relationships. Interestingly, the pathway prediction also depicted interconnected relationships between the receptors CCR2, CCR5, and CXCR2 (**Figure 2B**). Taken together, this suggested that neutrophils and monocytes may directly or indirectly play a part in the recruitment of one another during inflammation.

### 3.3 I3C prevents the influx of circulating inflammatory CCR2+ monocytes and CXCR2+ neutrophils in mice with ARDS

**Figure 3:**
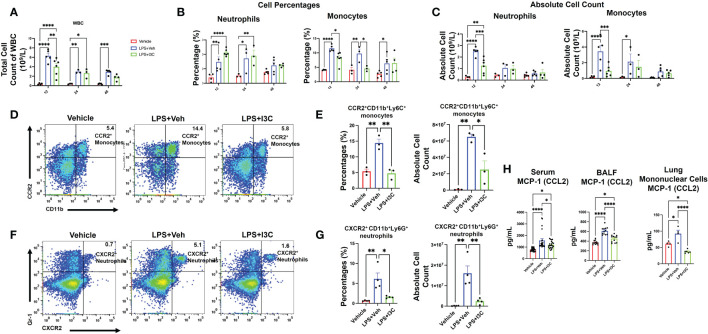
I3C decreases circulating inflammatory CCR2^+^ monocytes and CXCR2^+^ neutrophils in LPS-challenged mice. Mice were exposed to LPS to induce ARDS followed by treatment with I3C as described in the Methods. **(A)** Circulating white blood cell count at 12, 24, and 48 hours after exposure to LPS or LPS+I3C. **(B, C)** Cell percentages and absolute cell count of blood monocytes and neutrophils. **(D)** Representative flow cytometry plots of CCR2^+^ monocytes at 12hrs. **(E)** Bar graph of CCR2^+^ monocytes percentages and absolute cell count at 12hrs. **(F)** Representative flow cytometry plots of CXCR2^+^ neutrophils at 12hrs. **(G)** Bar graph of CXCR2^+^ neutrophils cell percentages and absolute cell count at 12hrs. **(H)** CCL2 protein level in bronchial lavage fluid, serum, and mononuclear cells supernatant detected by enzyme-linked immunosorbent assay. *p<0.05, **p<0.01, ***p<0.0002, ****p<0.0001.

To further investigate if the changes in myeloid cell profiles in the lungs during ARDS are associated with the changes in the monocyte and neutrophil populations in circulation during disease, we performed a differential white blood cell (WBC) count with blood samples. At 12 hours, there was an increase in the total count of WBCs in the blood of LPS+Veh *vs*. control mice (**Figure 3A**). Interestingly, I3C significantly decreased the total count of WBC in the blood of LPS-administered mice at 12 hours. At 24hrs, the WBC count in both LPS+Veh *vs*. LPS+I3C was lower than the 12 hour time point. However, WBC levels at 24 hours in LPS-challenged group was still significantly higher than baseline levels. Interestingly, the WBC levels in LPS+I3C group were returned almost to the baseline levels at 48 hours (**Figure 3A**).

Similar to the lungs, there was an increase in both monocytes and neutrophils in the blood of LPS-exposed animals *vs* Vehicle controls (**Figure 3B**). Furthermore, both neutrophil frequency and monocyte frequency were significantly increased at 12 and 24 hours in the LPS+Veh *vs* control group. At 48 hours, the neutrophil frequency was resolved, but there was still a significant increase in monocyte frequency at 48 hours in LPS+Veh *vs*. control group. Strikingly, there was a higher percentage of neutrophils in the blood of LPS+I3C mice *vs*. LPS+Veh and control mice at 12 hours and 24 hours. However, this may be due to I3C migration inhibitory capabilities, leading to an accumulation of neutrophils in the blood in diseased animals due to blocked cellular recruitment of the cells. However, at 48 hours, the frequency of neutrophils was similar across all treatment groups. We found that I3C attenuated the increased frequency of monocytes in LPS-exposed animals at 12 and 24 hours but monocyte percentages weren’t significantly different at 48 hours in LPS+I3C mice *vs* LPS+Veh (**Figure 3B**). Taken together, these data suggested that a downshift in the percentages of monocytes coincides with an upshift in the frequency of neutrophils in the blood. However, the absolute cell counts of monocytes and neutrophils in the blood across treatment groups showed that at 12 hours, I3C-treated mice had a significant reduction in both monocytes and neutrophils. At 24 hours, the neutrophil total count in LPS-exposed animals was returned to baseline, and at 48 hours, the monocyte total count in diseased animals was returned to baseline (**Figure 3C**).

Furthermore, we phenotyped the immune cells to identify whether monocytes and circulation neutrophils expressed CCR2, CCR5, or CXCR2 chemokines receptors associated with myeloid cell trafficking. Flow cytometric analyses of blood at 12 hours after LPS exposure showed a significant increase in CCR2+ monocytes and CXCR2+ neutrophils in LPS+Veh *vs* Vehicle-only group (**Figures 3D-G**; **Supplementary Figure 2**). Interestingly, there was no significant increase in any immune cell population expressing CCR5 across treatment groups at 12-48 hours (data not shown). In the presence of I3C, CCR2+ monocytes and CXCR2+ neutrophils in circulation were decreased in LPS-exposed mice (**Figures 3D-G**; **Supplementary Figure 2**). Thus, the data showed that the increase in CXCR2+ neutrophils and CCR2+ monocyte populations during disease at 12 hours was attenuated by I3C.

Notably, at 24 hours, in LPS+Veh group, CXCR2+ neutrophils and CCR2+ monocytes were resolved to the levels of LPS+I3C. At 48 hours, CXCR2+ neutrophils and CCR2+ monocytes in LPS+Veh group were returned to baseline (data not shown). Furthermore, monocyte chemoattractant protein-1 (CCL2), the chemoattractant ligand for the CCR2, was increased in the serum and BALF at 48 hours in LPS+Veh mice *vs* the control group. Strikingly, I3C treatment significantly lowered this increase in LPS-exposed animals. Additionally, mononuclear cells isolated from the lungs at 48 hours from the LPS-exposed animals were producing more CCL2 than the control group. We found that the production of CCL2 by mononuclear cells from the LPS+I3C group was significantly lower than in the LPS+Veh group and Vehicle-only group (**Figure 3H**). Taken together, these data suggested that I3C partially blocks the upshift in CCR2 monocytes and CXCR2 neutrophils in circulation during ARDS.

### 3.4 I3C partially blocks the influx of CCR2+ monocytes and the recruitment of CXCR2+ neutrophils in the lungs of mice with ARDS

**Figure 4:**
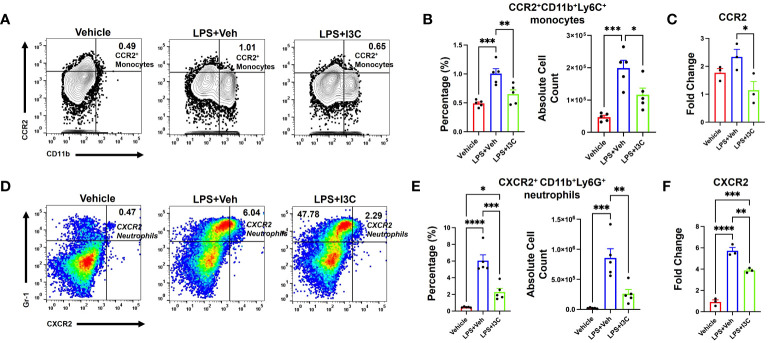
CCR2^+^ monocyte and CXCR2^+^ neutrophil populations increase following LPS exposure, which is attenuated by I3C. Mice were exposed to LPS to induce ARDS followed by treatment with I3C as described in the Methods. The cells isolated from the lungs were screened as follows: **(A)** Representative contour flow cytometry plots of CCR2^+^ monocytes seen in the lungs at 48hrs. **(B)** Bar graph of CCR2^+^ monocyte percentages and absolute cell numbers at 48hrs. **(C)** CCR2 mRNA expression in lung MNCs. **(D)** Representative flow cytometry plots of CXCR2^+^ neutrophils at 48hrs. **(E)** Bar graph of CXCR2^+^ neutrophil percentages and absolute cell numbers at 48hrs. **(F)** CXCR2 mRNA expression in whole lung cells. *p<0.05, **p<0.01, ***p<0.0002, ****p<0.0001.

At 48hrs following LPS exposure, we analyzed the lung immune cells to investigate the migration of CXCR2+ neutrophils and CCR2+ monocytes population, and the ability of I3C to prevent the increase in these specific cell types. LPS-challenge resulted in an increase in CCR2+ monocytes in the lungs (**Figures 4A, B**). Interestingly, I3C treatment caused a decrease in CCR2+ monocytes in the lungs and blood, findings consistent with a suppressed myeloid response (**Figures 3D, E**; **4A, B**). Further investigation into mononuclear cellular expression of the CCR2 gene showed that I3C downregulated the expression of the CCR2 gene in mononuclear cells of LPS-exposed mice (**Figure 4C**). Investigation into CXCR2+ neutrophils showed a significant increase in these cells in the lungs following LPS exposure and they were decreased in the LPS+I3C group (**Figures 4D, E**). The CXCR2 gene expression was decreased in the lung cells of LPS-exposed mice when treated with I3C (**Figure 4F**). These data further suggested that I3C suppresses CCR2+ monocytes and CXCR2+ neutrophil infiltration into the lungs during ARDS.

### 3.5 CXCR2+ neutrophil cell population frequency during ARDS is dependent on the CCR2+ monocytes

**Figure 5:**
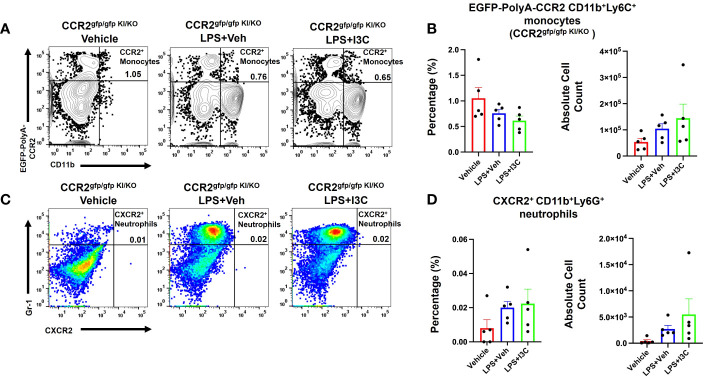
Cell percentages and numbers of CCR2^+^ monocyte and CXCR2^+^ neutrophil cell populations were not altered in CCR2^gfp/gfp^ knock-in/knock-out mice following LPS treatment. Mice were exposed to LPS to induce ARDS followed by treatment with I3C as described in the Methods. The cells isolated from the lungs were screened as follows: **(A)** Representative contour flow cytometry plots of EGFP-Poly-A-CCR2^+^ monocytes in the lungs of *CCR2^gfp/gfp^ KI/KO* mice at 48hrs. **(B)** Bar graph of EGFP-Poly-A-CCR2+ monocyte cell percentages and absolute cell numbers in *CCR2^gfp/gfp^ KI/KO* mice at 48hrs. **(C)** Representative flow cytometry plots of CXCR2^+^ neutrophils at 48hrs in the lungs. **(D)** Bar graph of CXCR2^+^ neutrophils cell percentages and absolute cell count at 48hrs in the lungs.

To investigate the role of infiltrating CCR2+ monocytes and their effects on CXCR2+ neutrophil recruitment during LPS-induced ARDS, we used a CCR2gfp/gfp knock-in/knock-out (KI/KO) mice. These mice have green fluorescent protein (EGFP) sequence followed by a polyadenylation signal inserted into the translation initiation site of CCR2 thereby abolishing its expression. In addition, CCR2gfp/gfp KI/KO mice lack peripheral blood Ly6chi monocytes (57). We observed that in CCR2gfp/gfp KI/KO mice, LPS+Veh group showed no significant increase in the percentage and total numbers of CCR2+ monocytes during LPS-induced ARDS when compared to the Vehicle group (**Figures 5A, B**). Also, this response was markedly attenuated when compared to the wild-type mice (**Figures 4A, B**). Also, CCR2gfp/gfp KI/KO mice treated with I3C showed no significant differences in the CCR2+ monocyte population when compared to the LPS-treated groups (**Figures 5A, B**). Next, we examined the CXCR2+ neutrophil response during ARDS in CCR2gfp/gfp KI/KO mice when CCR2 was not expressed on the cell surface of monocytes and macrophages. Strikingly, the LPS-induced ARDS-associated increase in the percentage and numbers of the CXCR2+ neutrophils in the lungs was markedly reduced in LPS-treated CCR2gfp/gfp KI/KO mice (**Figures 5C, D**) when compared to the WT mice (**Figures 4D, E**). I3C did not further significantly alter the CXCR2+ neutrophil population in LPS-treated CCR2gfp/gfp KI/KO mice (**Figures 5C, D**) suggesting that I3C-induced decrease in the CXCR2+ neutrophil population following LPS-exposure may result from a decrease in CCR2+ monocytes. Importantly, our studies suggested that infiltrating peripheral CCR2+ blood monocytes may be responsible for CXCR2+ neutrophil accumulation in the lungs during LPS-induced ARDS.

### 3.6 I3C-mediated decrease in the recruitment of CCR2+ monocytes and CXCR2+ neutrophils into the lungs during ARDS is dependent on AhR

**Figure 6:**
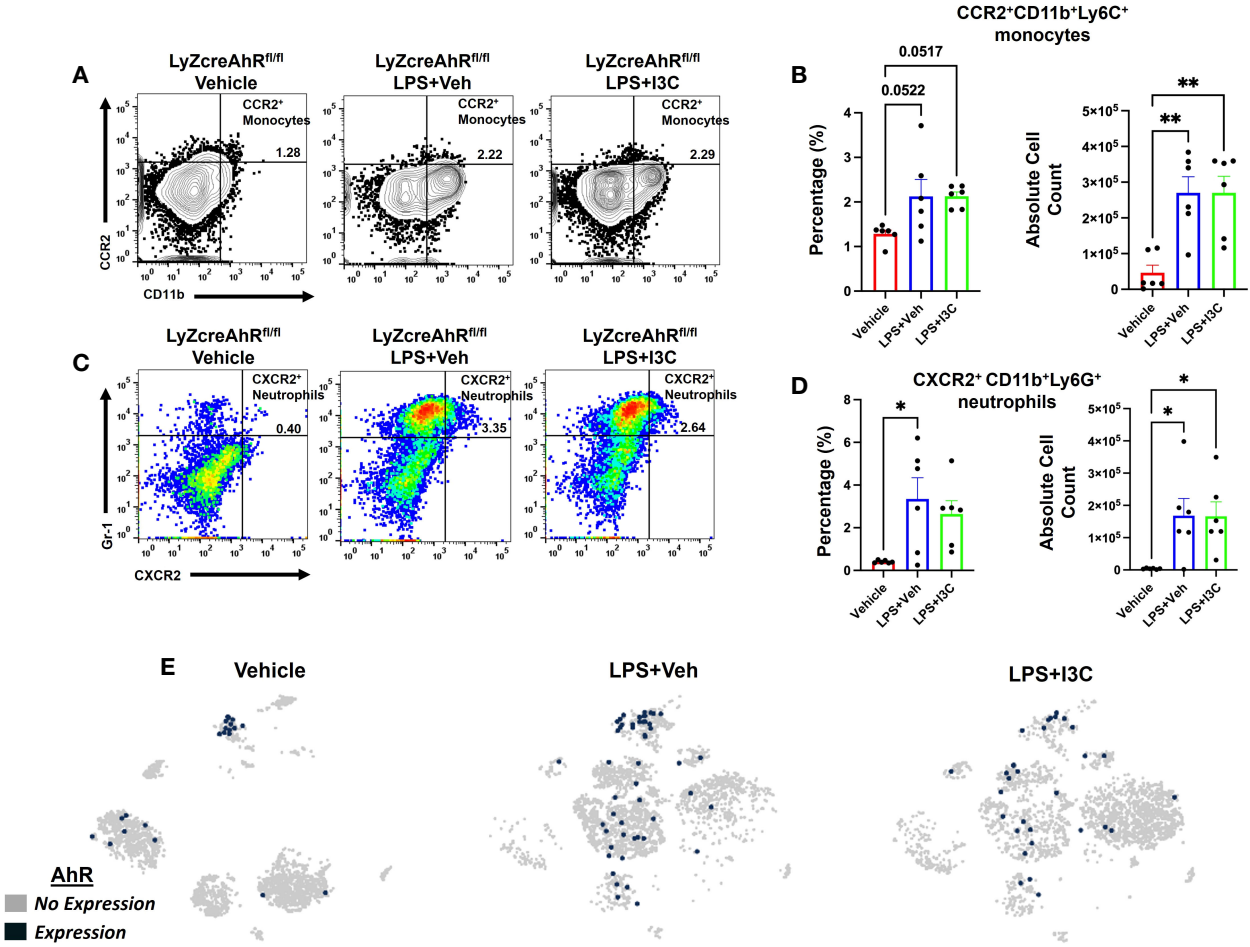
I3C-mediated attenuation of CCR2^+^ monocytes and CXCR2^+^ neutrophils in the lungs of LPS-exposed animals is dependent on the AhR. *LyZcreAhR^fl/fl^* mice were exposed to LPS to induce ARDS followed by treatment with I3C as described in the Methods. The cells isolated from the lungs were screened as follows: **(A)** Representative contour flow cytometry plots of CCR2^+^ monocytes at 48hrs. **(B)** Bar graph of CCR2^+^ monocyte percentages and absolute cell counts at 48hrs. **(C)** Representative flow cytometry plots of CXCR2^+^ neutrophils at 48hrs **(D)** Bar graph of CXCR2^+^ neutrophil percentages and absolute cell counts at 48hrs. **(E)** scRNAseq t-SNE heatmap expression of AhR. *p<0.05, **p<0.01.

Lastly, we investigated the role of AhR in I3C-mediated attenuation of CCR2+ monocyte and CXCR2+ neutrophil recruitment to lungs after LPS-challenge. To that end, we used LyZcreAhRfl/fl mice, which do not express AhR on myeloid linage cells. Like the wild-type C57BL/6 mice with ARDS, there was an increase in CCR2+ monocytes in the lungs of LyZcreAhRfl/fl mice following LPS administration (**Figures 6A, B**). Interestingly, unlike LPS+I3C-treated wild-type mice (**Figures 4A, B**), I3C-mediated attenuation of CCR2+ monocytes in the lungs did not occur in LPS-exposed LyZcreAhRfl/fl mice (**Figures 6A, B**). In addition, CXCR2+ neutrophil populations were not decreased in LPS-exposed LyZcreAhRfl/fl mice following I3C treatment (**Figures 6C, D**). Furthermore, scRNA-seq t-SNEs showed that AhR is expressed by macrophage and monocyte cell clusters expressing CCR2 in wild-type mice (**Figure 6E**). Collectively, these findings demonstrated that AhR is essential for an I3C-mediated decrease in CCR2+ monocytes and CXCR2+ neutrophils.

## 4 Discussion

ARDS is a life-threatening pulmonary disease that affects almost 200,000 patients annually. The disease carries a ~40% mortality rate due to limited effective therapeutic modalities and different etiologies that induce ARDS (13, 58–60). Recently, with the advent of COVID-19, the number of patients suffering from ARDS will continue to rise (4). One of the clinical features seen in patients suffering from SARS-CoV-2 induced COVID-19 is elevated numbers of neutrophils in the blood and respiratory tract (61, 62). Neutrophil abundance coincides with COVID-19 severity (58, 63, 64). Severe forms of COVID-19 lead to ARDS (3). Besides COVID-19, ARDS is frequently associated with bacterial infections and thus, LPS-induced ARDS is a significant mouse model to study the recruitment of neutrophils in the blood and respiratory tract during ARDS (10, 11). Similar to the influx of neutrophils seen in patients infected with SARS-CoV-2 and their abundance being associated with ARDS, the LPS-induced ARDS mouse model is a neutrophil-dependent lung injury model in which disease progression is dependent on the activation and transmigration of neutrophils to the alveolar airspace (11, 20).

Monocyte and neutrophil interactions are essential for host defense against foreign invaders and the clearance of pathogens. However, if not tightly regulated, these interactions can have detrimental consequences. Neutrophils are non-residential cells deployed in circulation during inflammation when residential cells release chemoattractant into the circulation (65, 66). During ARDS, tissue activation of resident macrophages results in an influx of neutrophils to the lungs (37, 39, 67). Neutrophils augment monocytes’ recruitment to the site of inflammation. It is also known that monocytes and neutrophils work in concert in response to pathogens (67, 68). However, both neutrophils and monocytes/macrophages have several different heterogeneous subsets, which may act in different ways thereby impacting the degree of inflammation and tissue injury. In the current study we demonstrated that CCR2+ monocytes recruit CXCR2+ neutrophils during LPS-mediated ARDS.

Under inflammatory conditions, CCR2, a chemokine receptor, plays a critical role in the trafficking of bone marrow-derived monocytes to the inflamed lungs, and contributing to the lung injury during ARDS. CCR2+ monocytes are a subset of monocytes that express the chemokine receptor. These cells can facilitate the recruitment of other immune cells like neutrophils via the direct recruitment of CXCR2 (37, 69). CXCR2, is a key chemokine receptor expressed on neutrophils and is responsible for the recruitment and trans-endothelial and trans-epithelial migration of neutrophils to the lung injury site, contributing to the lung injury severity (14, 70). Research has shown that CCR2+ monocytes can directly recruit CXCR2-expressing cells, such as neutrophils, to the lungs through a mechanism involving chemokine gradients and cell-cell interactions. One study investigated the role of CCR2+ monocytes in lung inflammation and found that these cells were responsible for recruiting neutrophils to the lungs in a mouse model of acute lung injury. The researchers demonstrated that CCR2+ monocytes produced chemokines such as CXCL1 and CXCL2, which are ligands for CXCR2, and these chemokines were crucial for the recruitment of CXCR2-expressing cells to the lungs (71). Furthermore, another study examined the role of CCR2+ monocytes in recruiting neutrophils during pneumonia. The researchers found that CCR2+ monocytes produced high levels of CXCL1, which directly recruited CXCR2-expressing neutrophils to the lungs, contributing to the host defense against bacterial infection (72). Interestingly, several studies have examined the clinical benefits of Navarixin (MK-7123/SCH 527123) and other CXCR2 inhibitors, a pharmacological intervention to block neutrophil recruitment to treat COVID-19. But side effects of taking the CXCR2 inhibitors include nasopharyngitis, headaches, and decreased neutrophil count, leaving the patient susceptible to respiratory infections and other infections (43–45, 73). In endotoxin-mediated ARDS, bone marrow-derived blood-borne monocytes expressing CCR2 are involved in amplifying neutrophilic alveolitis (37, 38, 74). The goal of the current study was to explore the relationship between CCR2+ monocytes and CXCR2+ neutrophils during LPS-mediated ARDS while also exploring the role of AhR activation in immune cell trafficking and lung injury. Consistent with previous studies (14, 37–39, 70, 73), we found that there’s an accumulation of CCR2+ monocytes and CXCR2+ neutrophils in the circulation and at the site of lung injury during LPS-mediated ARDS. The levels of chemokine CCL2 in the serum, BALF, and the production of CCL2 from mononuclear cells was increased in LPS-challenged animals. In the lungs of patients suffering from ARDS, CCL2 is upregulated (22, 67). The increase in CCL2 may be responsible for the increased frequency of CCR2+ monocytes during ARDS.

The AhR partially controls the activation and recruitment of CCR2+ monocytes during tissue injury (47, 75). Interestingly, I3C, a natural dietary AhR ligand, has been shown to inhibit CCL2 protein expression and CCR2+ monocyte recruitment to inflammatory sites (51). Consistent with these studies, we found that I3C decreased the recruitment and accumulation of CCR2+ monocytes under inflammatory conditions while regulating the frequency of CXCR2+ neutrophils in the circulation and the lungs during ARDS. We found that elevated counts of CCR2+ monocytes were associated with the elevated counts of CXCR2+ neutrophils in both the lungs and blood, suggesting a relationship between these myeloid cell subsets. Elevated counts of CCR2+ monocytes and CXCR2+ neutrophils in wild-type mice with ARDS significantly decreased following treatment with I3C but not abolished. Monocytes and neutrophils are involved in wound healing and recovery from tissue injury; therefore, abolishing monocytes and neutrophils or halting their recruitment may impact disease resolution and have dire consequences. This suggests that treatment with I3C may foster an environment for pathogen clearance and wound healing without the exaggerated response of CCR2+ monocytes and CXCR2+ neutrophils.

To examine if I3C directly affected CCR2+ blood-borne monocytes during ARDS, we used a CCR2gfp/gfp KI/KO mice, an EGFP knock-in, and CCR2 knock-out mice. CCR2gfp/gfp KI/KO mice lack peripheral blood Ly6chi monocytes. This allowed us to examine the residential macrophages that expressed the gene for CCR2 but not the receptor on the cell’s surface. Interestingly, LPS-challenge in CCR2gfp/gfp KI/KO mice did not exhibit significant increase in the CCR2+ monocytes or CXCR2+ neutrophils population, suggesting that CCR2 was essential for the recruitment of CCR2+ blood-borne monocytes and, in return, was essential for CXCR2+ neutrophils emigration in the lungs. In addition, I3C didn’t alter the CCR2 population or CXCR2+ neutrophils in CCR2gfp/gfp KI/KO mice. To further investigate if I3C-mediated attenuation of the accumulation of CCR2+ monocytes and CXCR2+ neutrophils was mediated through the AhR, we used LyZcreAhRfl/fl mice, with the loss of AhR on myeloid linage cells. These studies demonstrated that the infiltration of CCR2+ monocytes and CXCR2+ neutrophils was increased in LyZcreAhRfl/fl following LPS-challenge when compared to the controls. These results indicated that LPS was not causing the inflammatory response through the AhR on myeloid cells. However, unlike wild-type animals, I3C treatment did not decrease the frequency of CCR2+ monocytes and CXCR2+ neutrophils in the lungs of LyZcreAhRfl/fl diseased mice. This demonstrated that I3C-mediated attenuation of inflammatory myeloid cells in the lungs during ARDS was occurring through the ligation of the AhR. Thus, targeting the AhR ligand-receptor on myeloid linage cells may serve a potential intervention for ARDS.

## 5 Conclusions

In summary, disruption of CCR2 can interfere with monocyte-neutrophil cross-talk during ARDS. Our data support that CCR2+ monocytes are a prerequisite for the recruitment and accumulation of CXCR2+ neutrophils following LPS-induced ARDS. In addition, I3C can attenuate ARDS through activation of AhR I3leading to downregulation of CXCR2. Understanding the role of AhR ligands in the monocytes-neutrophil cross-talk may help with the development of novel therapeutics to treat patients suffering from ARDS

## Data availability statement

The data presented in this publication have been deposited in NCBI’s Gene Expression Omnibus and are accessible through the GEO Series accession number GSE224938, on the link https://www.ncbi.nlm.nih.gov/geo/query/acc.cgi?acc=GSE224938.

## Ethics statement

The animal study was approved by Institutional Animal Care and Use Committee. The study was conducted in accordance with the local legislation and institutional requirements.

## Author contributions

BH: Writing – review & editing, Writing – original draft, Visualization, Validation, Investigation, Formal analysis, Data curation, Conceptualization. KW: Writing – review & editing, Funding acquisition, Formal analysis, Data curation. AC: Writing – review & editing, Data curation. MN: Writing – review & editing, Supervision, Resources, Funding acquisition, Conceptualization. PN: Writing – review & editing, Supervision, Resources, Funding acquisition, Conceptualization.
