# Supplementary figures and images for: Indole-3-carbinol attenuates lipopolysaccharide-induced acute respiratory distress syndrome through activation of AhR: role of CCR2+ monocyte activation and recruitment in the regulation of CXCR2+ neutrophils in the lungs
Source: Front Immunol. 2024 Mar 26;15:1330373. doi: 10.3389/fimmu.2024.1330373 (PMC11002125; doi:10.3389/fimmu.2024.1330373)

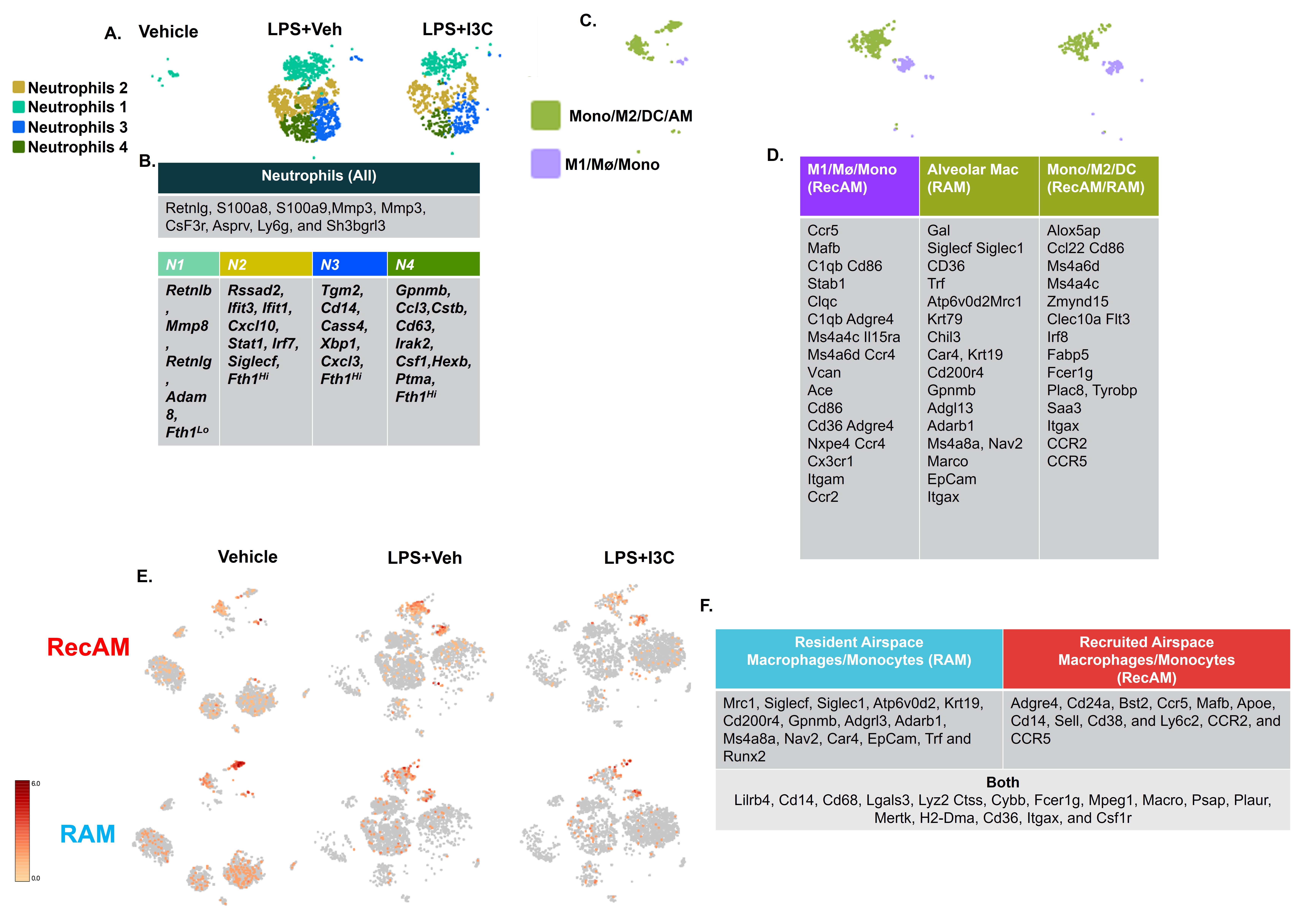

Supplement: Supplementary file 1 [file Image_1.jpeg]

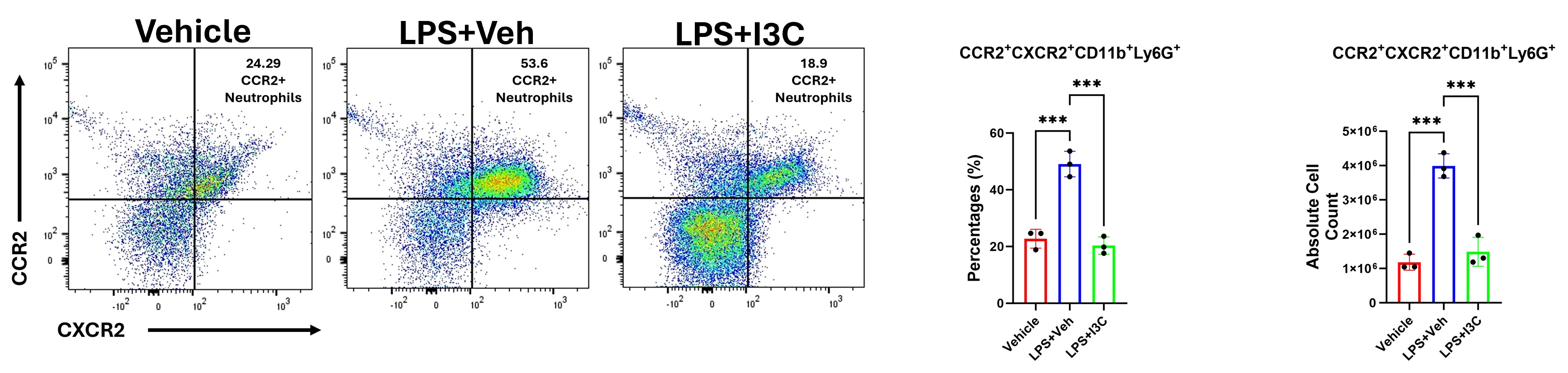

Supplement: Supplementary file 2 [file Image_2.jpeg]
